# Mechanisms of Microglial NLRP3 Inflammasome Activation by Methylmalonic Acid in Methylmalonic Acidemia

**DOI:** 10.1155/mi/1635641

**Published:** 2026-06-18

**Authors:** Shuqi Sun, Lu Sun, Yumei Su, Aihua Li, Lixin Hu, Qiliang Li

**Affiliations:** ^1^ Department of Clinical Laboratory Center, Beijing Children’s Hospital, Capital Medical University, National Center for Children’s Health, Beijing, 100045, China, ccmu.edu.cn

## Abstract

**Background:**

Microglial activation is a critical pathogenic mechanism of neurological damage in methylmalonic acidemia (MMA). The NOD‐like receptor pyrin domain containing 3 (NLRP3) inflammasome is closely associated with neuroinflammatory processes. However, the mechanism of NLRP3 inflammasome activation in microglia by methylmalonic acid (MMAcid) remains unclear.

**Methods:**

Mouse microglia BV2 cells and hippocampal neuronal HT22 cells were stimulated with different concentrations of MMAcid. Neuronal cells were treated with conditioned medium derived from differently treated microglia. Microscopy was used to observe morphological changes of cells. Cell counting kit‐8 (CCK‐8) assay was performed to assess cell viability (VB). In addition, the mRNA and protein levels were detected via qRT‐PCR, ELISA, and western blot. To further validate the mechanism, we used lentivirus infection and small interfering RNA (siRNA) transfection to overexpress (OE) and knockdown (KD) extracellular signal–regulated kinase (ERK) and analyzed the inflammatory pathway.

**Results:**

Direct exposure of neuronal cells to MMAcid significantly reduced cell VB, accompanied by morphological features suggestive of pyroptosis. MMAcid activated microglia, which was accompanied by increased expression of inflammatory molecules, activation of the ERK/nuclear factor‐κB (NF‐κB)/NLRP3 signaling pathway, and elevated secretion of interleukin‐1 beta (IL‐1β) and tumor necrosis factor‐α (TNF‐α). Neuronal cell VB decreased when cocultured with the supernatant from MMAcid‐treated microglia. Overexpression of ERK further enhanced microglial activation and potentiated MMA‐induced neuronal pyroptosis‐related signaling. Conversely, knocking down ERK expression in microglia reduced their activation by MMAcid and decreased cytokine secretion, but it did not completely reverse MMA‐induced neuronal damage.

**Conclusion:**

MMAcid is associated with microglial activation, accompanied by alterations in the ERK/NF‐κB/NLRP3 signaling pathway and increased release of inflammatory cytokines, which may contribute to neuronal pyroptosis–related changes.

## 1. Introduction

Methylmalonic acidemia (MMA) is the most common organic acidemia in children, caused by defects in methylmalonyl‐CoA mutase (MCM) or disruptions in cobalamin (cbl) metabolism [1, 2]. This leads to the accumulation of endogenous toxins, such as methylmalonic acid (MMAcid), resulting in multiorgan dysfunction, notably neurological damage [3–5]. While treatments such as vitamin B12 and L‐carnitine can manage acute crises, long‐term cognitive impairments often worsen, leading to severe consequences such as intellectual regression [6, 7]. Therefore, exploring the pathogenic mechanisms of neurological damage caused by MMA has become particularly crucial.

Previous studies have indicated that MMAcid‐induced neurological damage is characterized by neuroinflammation and neuronal apoptosis [8–10]. Microglia are intrinsic immune cells in the central nervous system (CNS). Normally, microglia remain in a resting state (M0), monitoring and regulating the microenvironment. In response to various stimuli, microglia can rapidly activate into M1 (neurotoxic) and M2 (neuroprotective) subtypes [11]. M1 polarization is characterized by the elevated expression of proinflammatory markers such as iNOS, whereas M2 polarization is typically indicated by upregulated anti‐inflammatory markers such as Arg1. Assessment of these representative markers helps delineate the microglial polarization status and associated inflammatory function. Activated M1 subtype microglia change their shape considerably into an “amoeba‐liked” structure and release many proinflammatory cytokines such as interleukin‐1 beta (IL‐1β) and tumor necrosis factor‐α (TNF‐α) mediated by multiple signaling cascades [12]. Overproduction of proinflammatory factors might exacerbate neuronal pyroptosis, contributing to the neurological damage caused by MMAcid [13, 14].

Extracellular signal–regulated kinase (ERK), as a member of the mitogen‐activated protein kinases (MAPKs) family, can activate the nuclear factor‐κB (NF‐κB) signaling pathway and further regulate NOD‐like receptor pyrin domain containing 3 (NLRP3) inflammasome activation [15–17]. The NLRP3 inflammasome has been extensively studied and is recognized as a pivotal inflammatory complex, particularly in microglia. The NLRP3 inflammasome [18–20] is composed of NLRP3, apoptosis‐related spot–like protein containing caspase recruitment domain (ASC), and cysteinyl aspartate specific proteinase‐1 (caspase‐1) precursor, acting as a key mediator of neuroinflammation and pyroptosis. After completing the NLRP3 inflammasome assembly, activated caspase‐1 cleaves proinflammatory cytokine precursors to produce mature cytokines and release them extracellularly [19, 21, 22]. However, the mechanism of NLRP3 inflammasome activation in microglia by MMAcid remains unclear.

In this study, we aim to examine the regulatory association of the ERK/NF‐κB/NLRP3 signaling pathway involved in microglial NLRP3 activation by MMAcid and the molecular mechanisms leading to neuronal pyroptosis. Our findings will provide potential intervention targets for the prevention and treatment of MMAcid‐induced neurotoxicity.

## 2. Materials and Methods

### 2.1. Cell Culture and Treatment

The mouse microglia BV2 and hippocampal neuronal HT22 cell line were obtained from BeNa Culture Collection (Jiangsu, China) and cultured in Dulbecco’s Modified Eagle Medium (DMEM) (Gibco, USA) supplemented with 10% fetal bovine serum (FBS) (Gibco, USA) and 100 U/mL penicillin/streptomycin (Hyclone, USA) at 37°C in a 5% CO_2_‐humidified atmosphere. When the cells reached ~80% confluence, they were digested with trypsin (Beyotime Biotechnology, Beijing, China) and passaged for further experiments. MMAcid (Sigma–Aldrich, USA) was diluted in the culture medium to various concentrations for cell stimulation. The supernatants of microglia treated under different conditions were collected and used to coculture neuronal cells. For MMAcid treatment, cells were exposed to 16 mmol/L MMAcid for the indicated times unless otherwise stated. Untreated cells cultured under normal conditions served as controls. To minimize potential confounding effects of pH variation, the pH of all MMAcid‐conditioned media was adjusted to ~7.3 using NaOH prior to cell treatment. In addition, the osmolarity of control medium and MMAcid‐conditioned medium was measured using a freezing point osmometer (Model 030, Gonotec Manufacturer). The osmolarity values were 299 mOsm/kg for the control medium and 312 mOsm/kg for the MMAcid‐conditioned medium.

### 2.2. Morphological Changes Observation

The BV2 microglia and HT22 neuronal cells were treated with MMAcid at concentrations of 8, 16, 24, 32, and 40 mmol/L for 1 or 3 days. After treatment, live‐cell microscopy was performed to monitor dynamic changes in the cell morphology.

### 2.3. Cell Counting Kit‐8 (CCK‐8) Cell Viability (VB) Assay

The cell VB was determined using a CCK‐8 (Beyotime Biotechnology, Beijing, China) as follows: Cells were plated at a density of 5 × 10^4^ cells/mL (100 µL/well) in 96‐well culture plates at 37°C with 5% CO_2_. Subsequently, the cells were treated with MMAcid at different concentrations or supernatants of microglia treated with different conditions for 1 or 3 days. Then, CCK‐8 solution (10 μL/well) was added to each well avoiding light exposure and incubated for 3 h at 37°C. The optical density (OD) value of each well was measured at 450 nm using a microplate reader (Rayto, Shenzhen, China). All assays were performed in triplicate.

Cell VB was calculated using the formula:

VB=OD value of the experimental group– OD value of the blank group/OD value of the control group– OD value of the blank group×100%.

### 2.4. ERK Overexpression Lentivirus Infection

To upregulate the expression of ERK in microglia, we used a lentivirus vector overexpressing ERK (JTS Scientific, Wuhan, China) to infect microglia. The lentiviral supernatant was collected and added to the microglial culture medium, followed by screening with puromycin.

### 2.5. Small Interfering RNA (siRNA) Transfection

To downregulate the expression of ERK in microglia, we used siRNA against ERK with the following sequence: forward 5′‐ AACCAUUGAGCAAAUGAAATT‐3′, reverse 5′‐UUUCAUUUGCUCAAUGGUUTT‐3′ (JTS Scientific, Wuhan, China). siRNA transfection was conducted according to the manufacturer’s instructions. In brief, the KEL transfection reagent and siRNA were mixed in DMEM, and microglia were incubated with the mixture for 6 h and then replaced to normal culture medium. After culturing for 48 h, the cells were used for further experiments.

### 2.6. Western Blot Analysis

Total proteins were extracted from cultured cells using RIPA lysis buffer and measured concentrations using the BCA protein detection kit (Thermo, USA). Equivalent amounts of extracted proteins (30 μg) were resolved by SDS‐PAGE (10% or 12%) and transferred onto PVDF membranes. GAPDH (Proteintech, Cat#10494‐1‐AP) was used as the single loading control for all targets because all proteins were resolved on the same gel and membrane. After blocking with 5% nonfat milk in TBST for 1.5 h, the membranes were incubated with the indicated primary antibodies at a dilution of 1:1000 overnight at 4°C, followed by the secondary antibodies at a dilution of 1:20,000 for 1 h at room temperature. After washing, the membranes were incubated with chemiluminescent HRP substrate (MeilunBio, Cat#MA0186) according to the manufacturer’s instructions and exposed using an enhanced chemiluminescence detection system (MiniChemi 610). The antibodies used in the study were as follows: toll‐like receptor‐4 (TLR‐4) (CST, Cat#38519), NLRP3 (Proteintech, Cat#19771), ERK (Proteintech, Cat#11257), phospho‐ERK (Proteintech, Cat#28733), NF‐κB (Proteintech, Cat#10745), phospho‐NF‐κB (CST, Cat#3033), caspase‐1 (Proteintech, Cat#22915), ASC (Proteintech, Cat#10500), IL‐1β (Proteintech, Cat#16806), TNF‐α (Proteintech, Cat#17590), GAPDH (Proteintech, Cat#10494), and goat antirabbit HRP secondary antibody (Beyotime, Cat# A0208).

### 2.7. RNA Extraction and Quantitative Real‐Time Polymerase Chain Reaction (PCR) Analysis

Total RNA was extracted from BV2 and HT22 cells with the Trizol reagent (Tiangen, Beijing, China) and was reverse‐transcribed to cDNA using PrimeScript RT Master Mix (Takara, Japan). Real‐time fluorescence quantitative PCR was performed using the TB Green Premix Ex Taq II (Takara, Japan) on the Gentier96E Real‐Time System (Tianlong, China). GAPDH served as the internal control. Gene expression was quantified using the 2^−ΔΔCt^ method. The primers were designed using Primer Blast. The sequences of primers used in this study are the follows: GAPDH, forward 5′‐TGTGTCCGTCGTGGATCTGA‐3′, reverse 5′‐TTGCTGTTGAAGTCGCAGGAG‐3′; IL‐1β, forward 5′‐TGACGGACCCCAAAAGATGAAGG‐3′, reverse 5′‐CCACGGGAAAGACACAGGTAGC‐3′; TNF‐α, forward 5′‐CAGGAGGGAGAACAGAAACTCCA‐3′, reverse 5′‐CCTGGTTGGCTGCTTGCTT‐3′; ERK, forward 5′‐ AAGACACAGCACCTCAGCAA‐3′, reverse 5′‐GTGTTCAGCAGGAGGTTGGA‐3′; and NLRP3, forward 5′‐ TATCCACTGCCGAGAGGTGA‐3′, reverse 5′‐TCTTGCACACTGGTGGGTTT‐3′.

### 2.8. ELISA Measurement

The cell supernatant was collected and centrifuged at 1000 g for 20 min to remove impurities and cell debris, and the supernatant was used for testing. The levels of IL‐1β and TNF‐α in the medium of BV2 cells were determined using an ELISA kit (Elabscience, Wuhan, China) according to the manufacturer’s instructions. The OD value of each sample was measured at a wavelength of 450 nm using a microplate reader (Rayto, Shenzhen, China). All assays were performed in triplicate.

### 2.9. Statistical Analysis

All experiments were performed with biological replicates defined as independent experiments conducted on different days using separately prepared cell cultures. Technical replicates represent repeated measurements within the same independent experiment. For all quantitative assays, the number of biological replicates (*n*) is indicated in the corresponding figure legends. Data are presented as mean ± standard deviation (SD). Before statistical calculations, continuous variables were first tested for normality and homogeneity of variance using the Shapiro–Wilk test. Parametric data were expressed as mean ± SD and analyzed using the *t*‐test. Nonparametric data were expressed as median and interquartile range (IQR) and analyzed using appropriate nonparametric tests. Comparisons among multiple groups were conducted using one‐way ANOVA followed by Tukey’s multiple comparisons test. In the normality test, *p* > 0.05 indicated a normal distribution. For all comparative analyses, *p* < 0.05 was considered statistically significant. All statistical analyses were performed using IBM SPSS 26.0 software and GraphPad Prism 9.5.

## 3. Results

### 3.1. MMAcid Activates Microglia

**Figure 1:**
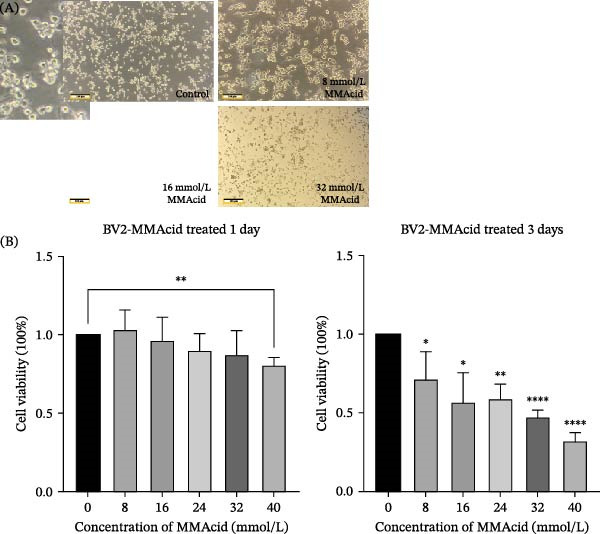
MMAcid activates microglia directly. (A) Effects of different concentrations of methylmalonic acid on the morphological changes in microglia for 3 days. Scale bar = 100 µm. (B) Effects of different concentrations of methylmalonic acid on the cell viability in microglia; MMAcid, methylmalonic acid;  ^∗^ *p* < 0.05,  ^∗∗^ *p* < 0.01, and  ^∗∗∗∗^ *p* < 0.0001, compared with the control group treated with 0 mmol/L MMAcid. Data are presented as mean ± SD (*n* = 3 biological replicates).

Previous experiments have shown that transient exposure to MMAcid may alter the redox state and induce neuroinflammatory/apoptotic processes, as well as glial activation during critical periods of brain development [10, 13]. To explore the effect of MMAcid on microglia, we treated BV2 microglia with various concentrations of MMAcid; then, we observed microglial morphological changes under a microscope and assessed cell VB using the CCK‐8 assay. The results indicated that after 1 day of treatment, the microglia exhibited an activated M2 state characterized by enlarged cell bodies and the extension of short pseudopods. Microglial cell VB decreased significantly (*p* < 0.01) when MMAcid reached a high concentration of 40 mmol/L. Research has reported a transition between M1/M2 phenotypes, with varying effects on disease progression at different stages [23–25]. When the treatment duration was extended to 3 days, the impact of MMAcid on BV2 cells became significant. At 8 mmol/L MMAcid, BV2 cells were activated to extend short pseudopods. At 16 mmol/L MMAcid, the microglia exhibited enlarged cell bodies and thickened branches with multiple pseudopods, resembling the “amoeboid‐liked” structure of M1 microglia. At 32 mmol/L MMAcid, BV2 cells elongated into a “drawn‐out” state, with numerous vacuoles in the cytoplasm and incomplete cell walls. Some microglia exhibited nuclear exclusion and apoptosis (Figure 1A). To further define the microglial polarization status, we performed RT‐qPCR to detect representative M1 marker iNOS and M2 marker Arg1 in BV2 cells. MMAcid treatment significantly upregulated iNOS mRNA expression, while Arg1 expression was not correspondingly increased, indicating a shift toward an M1‐like proinflammatory phenotype (Figure S1). Cell VB of microglia treated with MMAcid concentrations of 8, 16, 24, 32, and 40 mmol/L was determined to be 0.72 ± 0.17, 0.57 ± 0.18, 0.59 ± 0.09, 0.48 ± 0.04, and 0.32 ± 0.05, respectively. Those results indicated that microglial cell VB decreased with increasing MMAcid concentration, displaying a significant concentration‐dependent trend (Figure 1B).

### 3.2. MMAcid‐Induced Microglial Activation Involves NLRP3 Inflammasome Signaling

**Figure 2:**
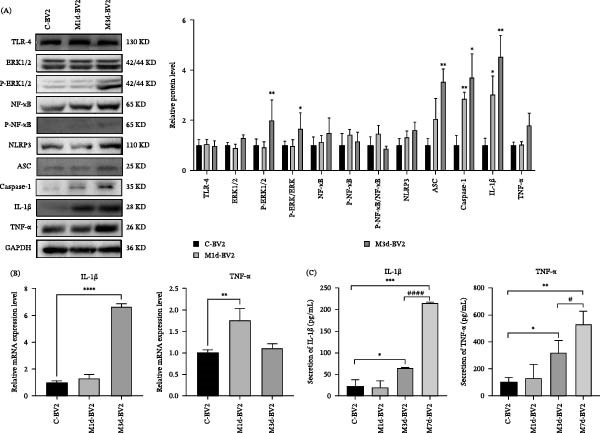
MMAcid enhances NLRP3 inflammasome‐related signaling. (A) The protein expression of inflammatory molecules in BV2 cells. (B) The mRNA expression of IL‐1β and TNF‐α in BV2 cells. (C) The secretion of IL‐1β and TNF‐α in BV2 cells’ supernatant. C‐BV2: control group BV2 cells under normal culture conditions; M1d‐BV2: BV2 cells treated with 16 mmol/L methylmalonic acid for 1 day; M3d‐BV2: BV2 cells treated with 16 mmol/L methylmalonic acid for 3 days; and M7d‐BV2: BV2 cells treated with 16 mmol/L methylmalonic acid for 7 days. GAPDH served as the common loading control for all targets. Data are presented as mean ± SD (*n* = 3 biological replicates). ( ^∗^ *p* < 0.05;  ^∗∗^ *p* < 0.01;  ^∗∗∗^ *p* < 0.001,  ^∗∗∗∗^ *p* < 0.0001, compared with the control group; ^#^ *p* < 0.05; ^####^ *p* < 0.0001, compared to the M3d‐BV2 group).

Preliminary titration experiments across a range of MMAcid concentrations (8–40 mmol/L) revealed that 16 mmol/L MMAcid consistently triggered robust morphological activation of BV2 microglia without causing excessive cell death. Therefore, this concentration was used in subsequent experiments unless otherwise specified. To further investigate the potential mechanism of activation of microglia by MMAcid, we conducted western blot analysis. The results revealed that after treatment of 16 mmol/L MMAcid, the protein expression levels of the molecules associated with the ERK/NF‐κB/NLRP3 signaling pathway increased to varying degrees. Interestingly, the expression of these molecules showed a time‐dependent increase with prolonged MMAcid treatment. Specifically, after 1 day of MMAcid treatment, proteins associated with inflammation and pyroptosis, such as caspase‐1 and IL‐1β, were significantly increased compared to normally cultured microglia. After 3 days of treatment, the levels of phosphorylated ERK, caspase‐1, ASC, and IL‐1β were dramatically elevated compared to the first day and baseline levels in untreated microglia (Figure 2A).

It has been reported that activation of the NLRP3 inflammasome promotes the release of mature cytokines [26]. To characterize the expression of IL‐1β and TNF‐α under MMAcid treatment, we examined mRNA levels by RT‐qPCR (1 and 3 days of MMAcid treatment) and quantified cytokine secretion in the culture supernatant by ELISA (1, 3, and 7 days of MMAcid treatment).

Because patients with MMA are chronically exposed to elevated MMA in the CNS, we included a 7‐day ELISA measurement to approximate the sustained metabolic stress that microglia may face in vitro. After 1 day of MMAcid treatment, the mRNA expression level of TNF‐α increased significantly. After 3 days of MMAcid treatment, IL‐1β mRNA expression levels were substantially elevated compared to the control group (Figure 2B, *p* < 0.05). As secretory proteins, the levels of IL‐1β and TNF‐α in the BV2 cell supernatant significantly increased after 3 days of MMAcid treatment (*p* < 0.05). After 7 days of treatment, these cytokine levels further increased and were significantly higher than those after 3 days (Figure 2C, *p* < 0.05).

### 3.3. Activation of Microglia by MMAcid Exacerbates MMAcid‐Induced Neuronal Damage

**Figure 3:**
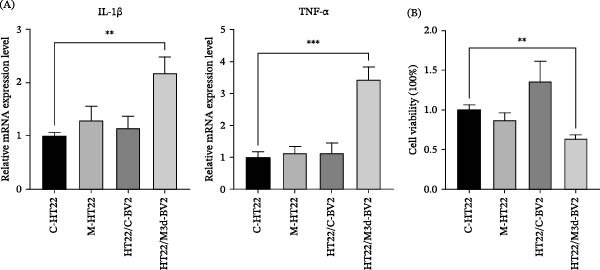
Activation of microglia by MMAcid exacerbates MMAcid‐induced neuronal damage. (A) The impact of MMAcid and MMAcid‐induced microglial activation on the mRNA expression of IL‐1β and TNF‐α in neuronal cells. (B) The effect of MMAcid and MMAcid‐induced microglial activation on the cell viability of HT22 neuronal cells. C‐HT22: control group of HT22 cells cultured under normal conditions; M‐HT22: HT22 cells directly treated with methylmalonic acid; HT22/C‐BV2: HT22 cells cocultured with the supernatant of normal cultured BV2 cells; and HT22/M3d‐BV2: HT22 cells cocultured with the supernatant of BV2 cells treated with methylmalonic acid for 3 days. Data are presented as mean ± SD (*n* = 3 biological replicates). ( ^∗∗^ *p* < 0.01 and  ^∗∗∗^ *p* < 0.001 compared to the control group).

To further explore the direct effects of MMAcid on HT22 neuronal cells and the synergistic effect of MMAcid‐induced microglial activation on HT22 neuronal cells, we conducted three experimental groups: HT22 cells directly treated with MMAcid (M‐HT22), HT22 cells cocultured with the supernatant of untreated BV2 cells (HT22/C‐BV2), and HT22 cells cocultured with the supernatant of BV2 cells treated with MMAcid for 3 days (HT22/M3d‐BV2). The normally cultured HT22 cells served as the control group. We used RT‐qPCR to measure the relative mRNA expression levels of IL‐1β and TNF‐α in HT22 cells under different treatments and performed the CCK‐8 assay to assess neuronal cell VB. The results showed that direct treatment with MMAcid slightly reduced the VB of HT22 cells without affecting IL‐1β and TNF‐α mRNA levels. However, coculture with the supernatant from MMAcid‐treated BV2 cells significantly increased IL‐1β and TNF‐α mRNA levels in HT22 cells (Figure 3A) and decreased cell VB (Figure 3B, *p* < 0.01).

We further examined the GSDMD‐N expression by western blot. The results demonstrated that MMAcid treatment significantly increased GSDMD cleavage, suggesting involvement of pyroptosis‐related signaling, while conditioned medium from MMAcid‐treated microglia further elevated GSDMD‐N levels. These data are presented in Figure S2.

### 3.4. Validation of ERK/NLRP3 Signaling Pathway of Microglial Activation and the Effects on Neuronal Damage Induced by MMAcid

As a member of the MAPK family, the ERK signaling pathway is implicated in modulating the activation state of microglia and the release of inflammatory mediators, thereby influencing the degree and duration of neuroinflammation. In this study, we used lentivirus infection and siRNA transfection to overexpress (OE group) and downregulate expression (KD group) of the ERK gene in BV2 cells. BV2 cells were then treated with 16 mmol/L MMAcid for 3 days.

#### 3.4.1. The Effects of ERK Gene Overexpression in Microglia on ERK/NLRP3 Signaling Pathway and Neuronal Damage

**Figure 4:**
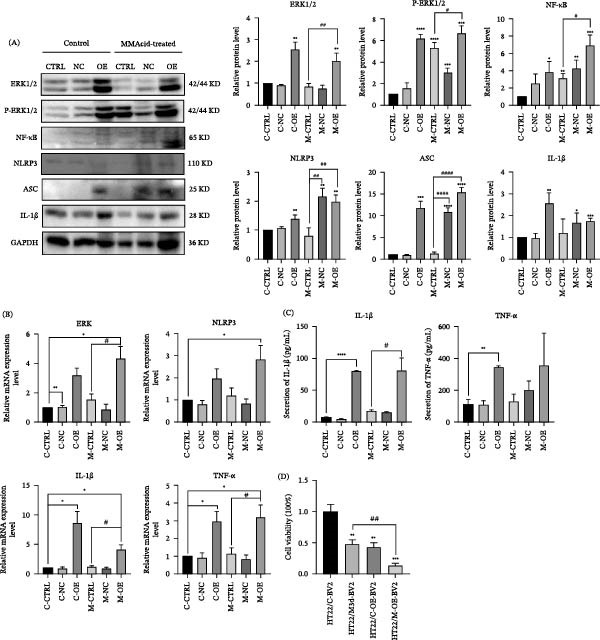
Overexpressing the ERK gene activates microglia and exacerbates neuronal cell damage induced by MMAcid. (A) Protein expression of inflammatory molecules in BV2 cells overexpressing ERK gene after MMAcid treatment. (B) mRNA expression of ERK, NLRP3, IL‐1β, and TNF‐α in BV2 cells overexpressing ERK gene after MMAcid treatment. (C) Secretion of IL‐1β and TNF‐α in BV2 cells overexpressing ERK gene after MMAcid treatment. (D) Cell viability of HT22 neuronal cells cocultured with culture supernatants from BV2 cells with different treatments. CTRL: BV2 cells in the control group cultured under normal conditions; NC: BV2 cells infected with empty lentivirus; and OE: BV2 cells overexpressing the ERK gene. C‐CTRL: untreated BV2 cells in the control group cultured under normal conditions; C‐NC: BV2 cells infected with empty lentivirus and cultured under normal conditions; C‐OE: BV2 cells overexpressing the ERK gene cultured under normal conditions; M‐CTRL: BV2 cells treated with MMAcid for 3 days; M‐NC: BV2 cells infected with empty lentivirus and treated with MMAcid for 3 days; and M‐OE: BV2 cells overexpressing the ERK gene treated with MMAcid for 3 days. HT22/C‐BV2: HT22 neuronal cells cocultured with culture supernatant from BV2 cells cultured normally; HT22/M3d‐BV2: HT22 neuronal cells cocultured with culture supernatant from BV2 cells treated with MMAcid for 3 days; HT22/C‐OE‐BV2: HT22 neuronal cells cocultured with culture supernatant from BV2 cells overexpressing the ERK gene cultured under normal conditions; and HT22/M‐OE‐BV2: HT22 neuronal cells cocultured with culture supernatant from BV2 cells overexpressing the ERK gene treated with MMAcid for 3 days. GAPDH served as the common loading control for all targets. Data are presented as mean ± SD (*n* = 3 biological replicates). ( ^∗^ *p* < 0.05,  ^∗∗^ *p* < 0.01, and  ^∗∗∗∗^ *p* < 0.0001 compared to the control group; ^#^ *p* < 0.05 and ^##^ *p* < 0.01 compared to the M‐CTRL group).

We used lentivirus to infect BV2 cells to OE the ERK gene. Microscopic images of microglia were taken under white light and fluorescence microscopy to verify the infection efficiency. Cells with green fluorescence indicated successful infection (Figure S3). Western blot analysis revealed that treatment with MMAcid alone significantly increased the phosphorylation of ERK, as well as the expression of NF‐κB compared with the control group. Furthermore, ERK overexpression in BV2 cells elevated inflammatory molecules even without MMAcid stimulation, suggesting that ERK activation itself is associated with a basal proinflammatory state. When ERK‐overexpressing cells were exposed to MMAcid, the levels of phosphorylated ERK and NF‐κB and downstream inflammasome‐related proteins were further amplified, indicating a synergistic enhancement of inflammatory signaling (Figure 4A). Moreover, ERK overexpression further enhanced iNOS expression and suppressed Arg1 expression, consistent with amplified M1‐like proinflammatory polarization (Figure S1). Under normal culture conditions, the mRNA expression levels of IL‐1β and TNF‐α in BV2 cells overexpressing the ERK gene (C‐OE group) significantly increased compared to the control group (C‐CTRL group). Under MMAcid treatment, BV2 cells overexpressing the ERK gene (M‐OE group) showed further increases in the mRNA expression of IL‐1β and TNF‐α, which were not only significantly different from those in BV2 cells cultured under normal conditions (C‐CTRL group) but also significantly higher than those in BV2 cells treated with MMAcid (M‐CTRL group). The mRNA expression of NLRP3 in BV2 cells overexpressing the ERK gene (M‐OE group) also increased, showing significant differences compared to the control group (C‐CTRL group) (Figure 4B). Similar results were obtained through ELISA. Under normal culture conditions, BV2 cells overexpressing the ERK gene secreted increased levels of inflammatory cytokines. Under MMAcid treatment, BV2 cells overexpressing the ERK gene secreted even higher levels of cytokines compared to BV2 cells treated with MMAcid alone (Figure 4C).

BV2 cells overexpressing the ERK gene secreted increased levels of inflammatory cytokines without any treatment, leading to a significant decrease in the VB of HT22 neuronal cells (HT22/C‐OE‐BV2) compared to the control group. Furthermore, under MMAcid treatment, the secretion of cytokines further increased in ERK overexpression BV2 cells, resulting in a significant decrease in the VB of HT22 cells cocultured with their culture supernatant. This decrease was significant compared to the control group and also markedly lower than HT22 cells cocultured with the culture supernatant of BV2 cells treated with MMAcid alone (HT22/M3d‐BV2) (Figure 4D). These results indicated that both MMAcid intervention in BV2 cells and overexpression of the ERK gene lead to the secretion of large amounts of inflammatory cytokines by activated microglial cells. When BV2 cells overexpressing the ERK gene are additionally subjected to MMAcid treatment, cytokine secretion was further enhanced, and microglia secreted cytokines and exacerbated neuronal damage in HT22 cells.

#### 3.4.2. The Effects of Knockdown (KD) Expression of ERK Gene in Microglia on ERK/NLRP3 Signaling Pathway and Neuronal Damage

**Figure 5:**
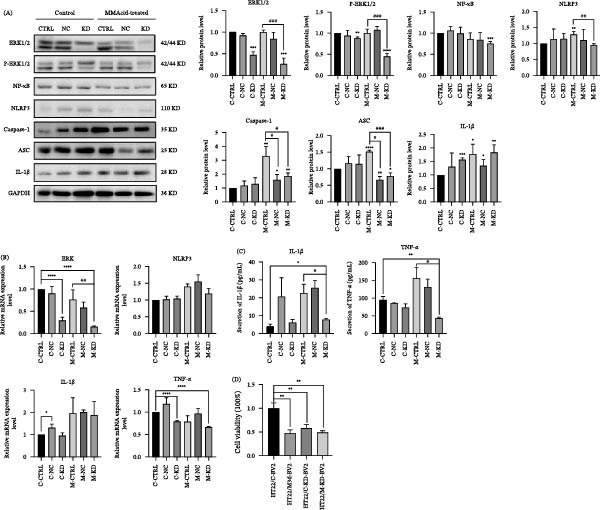
Downregulating the expression of ERK gene can reduce the microglial activation induced by MMAcid. (A) Protein expression of inflammatory molecules in BV2 cells with ERK gene knockdown after MMAcid treatment. (B) mRNA expression of ERK, NLRP3, IL‐1β, and TNF‐α in BV2 cells with ERK gene knockdown after MMAcid treatment. (C) Secretion of IL‐1β and TNF‐α in BV2 cells with ERK gene knockdown after MMAcid treatment. (D) Cell viability of HT22 neuronal cells cocultured with culture supernatants from BV2 cells with different treatments. CTRL: BV2 cells under normal culture conditions; NC: BV2 cells transfected with negative control siRNA; KD: BV2 cells with ERK gene knockdown. C‐CTRL: untreated BV2 cells under normal culture conditions; C‐NC: BV2 cells transfected with negative siRNA under normal culture conditions; C‐KD: BV2 cells with ERK gene knockdown under normal culture conditions; M‐CTRL: BV2 cells treated with MMAcid for 3 days; M‐NC: BV2 cells transfected with negative siRNA and treated with MMAcid for 3 days; M‐KD: BV2 cells with ERK gene knockdown treated with MMAcid for 3 days; HT22/C‐BV2: control group; HT22 neuronal cells cocultured with culture supernatant from BV2 cells cultured under normal conditions; HT22/M3d‐BV2: HT22 neuronal cells cocultured with culture supernatant from BV2 cells treated with MMAcid for 3 days; HT22/C‐KD‐BV2: HT22 neuronal cells cocultured with culture supernatant from BV2 cells with ERK gene knockdown cultured under normal conditions; and HT22/M‐KD‐BV2: HT22 neuronal cells cocultured with culture supernatant from BV2 cells with ERK gene knockdown treated with MMAcid for 3 days. GAPDH served as the common loading control for all targets. Data are presented as mean ± SD (*n* = 3 biological replicates). ( ^∗^ *p* < 0.05,  ^∗∗^ *p* < 0.01,  ^∗∗∗∗^ *p* < 0.0001 compared to the control group; ^#^ *p* < 0.05 and ^##^ *p* < 0.01 compared to the M‐CTRL group).

As shown in Figure 5A, with MMAcid treatment, BV2 cells with ERK gene KD (M‐KD group) showed significant lower protein expression levels of inflammatory markers such as P‐ERK, NLRP3, caspase‐1, and ASC compared to normal BV2 cells (M‐CTRL group). Additionally, ERK KD partially reduced iNOS expression and tended to restore Arg1 levels, supporting a regulatory role of ERK in modulating MMAcid‐induced M1‐like microglial polarization.

KD of the ERK gene resulted in a significant decrease in the mRNA expression of TNF‐α in BV2 cells under both normal culture conditions and MMAcid treatment (Figure 5B, *p* < 0.0001). The secretion levels of cytokines IL‐1β and TNF‐α in the culture supernatant of BV2 cells were measured after different treatments. Under normal culture conditions, siRNA transfection did not increase cytokine secretion in BV2 cells. However, under MMAcid treatment, BV2 cells with ERK gene KD (M‐KD group) showed a significant decrease in the secretion of IL‐1β and TNF‐α compared to BV2 cells expressing normal levels of ERK (M‐CTRL group) (Figure 5C). These results indicate that knocking down the ERK gene reduces the expression of inflammatory pathway molecules to some extent.

We then cocultured HT22 neuronal cells with the culture supernatant of BV2 cells subjected to different treatments and measured the VB of HT22 cells. The results showed that under MMAcid treatment, although cytokine secretion decreased slightly in BV2 cells with ERK gene KD, HT22 cell VB was still compromised (Figure 5D). Knocking down the ERK gene did not completely reverse the synergistic effect of MMAcid‐activated microglia on neuronal damage.

## 4. Discussion

Neurological damage is the most common symptom in MMA patients [27], yet the molecular mechanisms underlying MMAcid‐induced neurologic damage remain underexplored. Through establishing MMA animal model, previous research [10] revealed that continuous MMAcid stimulation to cerebral tissue can activate microglia and further trigger oxidative stress and inflammatory responses, which subsequently lead to neuronal apoptosis and brain damage. In this study, we investigated the inflammatory pathways of microglia activated by MMAcid through establishing a cell model. Additionally, we explored the role and mechanism of microglia in neuronal injury induced by MMA through cocultivation of MMA‐activated microglial medium with neuronal cells in vitro. To validate the signaling pathway involved, we employed ERK gene knocking down and overexpressing in microglia. Our study indicates that MMA‐induced microglial activation is linked to the ERK/NF‐κB/NLRP3 pathway, accompanied by increased secretion of inflammatory cytokines and associated neuronal damage.

Microglia are the intrinsic immune cells of the CNS and play crucial roles in regulating neuronal numbers and responding to various stimulations [28–30]. Microglia are highly sensitive to being activated in the CNS and rapidly initiate immune response in the presence of infection, injury, or other harmful stimuli [31]. Currently, microglia are categorized into two states [31, 32]: the resting state (M0) and the activated state, further divided into M1 and M2 subtypes. The polarization process towards the M1 subtype is mediated by TLR‐4 and involves activation of the ERK/NF‐κB/NLRP3 signaling pathway. M1 microglia are considered neurotoxic, releasing inflammatory factors that damage neurons and induce pyroptosis, typically seen in chronic injuries [33, 34]. In contrast, M2 microglia are neuroprotective, releasing anti‐inflammatory factors that promote neuronal health, particularly during acute injury periods [35, 36]. In this study, microglia cultured under normal conditions represented the resting state (M0). Following treatment of BV2 microglia with various concentrations of MMAcid for different durations, distinct cellular responses were observed. After 1 day of low‐concentration MMAcid treatment, microglia exhibited a fuller and rounder morphology, displaying the M2 microglial characteristics. However, after 3 days of MMAcid treatment, microglia exhibited enlarged cell bodies and thickened branches, resembling the “amoeboid‐liked” structure characteristic of M1 microglia [37]. MMAcid‐treated microglia displayed typical proinflammatory features characterized by significantly upregulated M1 marker iNOS without a corresponding increase in the M2 marker Arg1, suggesting a tendency toward an M1‐like polarization profile. The lack of obvious changes in M2‐related gene expression may be explained by the nature of the stimulus: MMAcid acts as a metabolic stressor rather than a strong canonical M1‐polarizing stimulus (e.g., LPS plus IFN‐γ). M1 polarization is known to rely on glycolysis and ERK/NF‐κB signaling, which are consistent with the pathways activated by MMAcid, whereas M2 polarization depends on oxidative metabolism and STAT6 signaling, which may not be substantially affected under our experimental conditions. Thus, MMAcid preferentially promotes M1‐related transcriptional programs without directly inhibiting M2‐related pathways. Furthermore, microglial cell VB decreased with increasing MMAcid concentrations, demonstrating a concentration‐dependent pattern.

Previous research indicated that neurological symptoms in MMA patients can be attributed to brain damage, such as neuronal degeneration in the striatum, cortical atrophy, and lesions in the basal ganglia, particularly the globus pallidus [38, 39]. Stimulation by MMAcid induces ERK phosphorylation, which is associated with microglial activation and increased inflammatory responses. In this study, treatment with 16 mmol/L MMAcid resulted in increasing protein expression levels of inflammation‐related molecules in microglia, including NLRP3, NF‐κB, phosphorylated NF‐κB, ERK, phosphorylated ERK, caspase‐1, ASC, IL‐1β, and TNF‐α. Furthermore, prolonged treatment further elevated the protein expression levels of these molecules, indicating a time‐dependent effect. These findings indicate that MMAcid is associated with increased ERK phosphorylation and NF‐κB activation in microglia, accompanied by NLRP3 inflammasome activation and increased caspase‐1 expression, as well as elevated IL‐1β and TNF‐α production. To further validate the mechanism, we used siRNA transfection and lentivirus infection to KD and OE ERK and analyzed the inflammatory pathway. ERK overexpression markedly enhanced the phosphorylation of ERK and upregulated NF‐κB and NLRP3 inflammasome‐related proteins, indicating that ERK activation amplifies inflammatory signaling. In contrast, ERK gene KD alleviates the inflammatory activation induced by MMAcid, supporting that ERK signaling contributes to the regulation of NF‐κB/NLRP3‐associated inflammatory signaling. Moreover, ERK overexpression alone slightly increased basal inflammatory markers, whereas ERK KD suppressed cytokine expression even after MMAcid stimulation. These results suggest that ERK acts not only as a mediator but also as an amplifier of microglial responses to metabolic stress.

Pyroptosis [40–42] is a novel form of programmed cell death associated with inflammation, combining features of both apoptosis and necrosis. Unlike conventional necrosis, pyroptosis and its resulting inflammation are controlled processes [43]. Currently, the prevailing perspective suggests that microglia play a detrimental role in neurogenic diseases [44] when the CNS encounters stimuli or damage. M1 microglia, as sentinel immune cells, initiate an immune response by releasing abundant neurotoxins and inflammatory factors, contributing to neuronal pyroptosis [45, 46]. In our study, we cocultured HT22 neuronal cells with supernatants from microglia subjected to various treatments. Our results demonstrated that while MMAcid had a minimal impact on neuronal cell VB, it did not affect the mRNA expression levels of IL‐1β and TNF‐α. Furthermore, coculturing neurons with MMAcid‐treated microglial supernatants for 72 h significantly upregulated the mRNA expression levels of IL‐1β and TNF‐α, accompanied by a notable decrease in cell VB. Those results suggest that microglia activated by MMAcid can induce neuronal cells to produce abundant inflammatory factors, triggering a cascade of inflammatory responses, ultimately leading to neuronal pyroptosis.

Furthermore, we found that ERK KD modestly decreased the secretion of cytokines in microglia but did not fully reverse the synergistic effect of MMAcid‐activated microglia on neuronal damage. This suggested that ERK signaling contributes to the regulation of microglial NLRP3–associated inflammatory responses, but it may not be the only mechanism. Moreover, after MMAcid treatment, microglia overexpressing ERK showed increased cytokine secretion, accompanied by enhanced neuronal pyroptosis–related changes. This effect may be associated with the combined effects of MMAcid treatment and ERK overexpression, leading to a more robust inflammatory response in microglia.

Several limitations of this study should be acknowledged. All experiments were performed in BV2 microglial cells, which may not fully reflect the responses of primary microglia or the complex in vivo environment. Although a 7‐day MMAcid treatment ELISA time point was included to approximate chronic MMAcid exposure, prolonged culture can introduce nonspecific stress, and these data should be interpreted cautiously. Additionally, microglial polarization was evaluated based on representative transcriptional markers (iNOS and Arg1) rather than comprehensive functional assays, thus reflecting a polarization tendency rather than a fully defined functional phenotype. Moreover, we focused on the regulatory association of the ERK/NF‐κB/NLRP3 pathway but did not perform pharmacological inhibition or rescue experiments to establish definitive causality. Causality remains to be confirmed in future studies. In addition, although relatively high concentrations of MMAcid were used in this study, pH and osmolarity were carefully controlled within physiological ranges, suggesting that the observed cellular responses are unlikely to be attributable to nonspecific physicochemical effects. Future work will use primary microglia, coculture systems, and animal models to confirm and extend these findings.

## 5. Conclusion

MMAcid is associated with microglial activation, accompanied by alterations in the ERK/NF‐κB/NLRP3 signaling pathway and increased release of inflammatory cytokines, which may contribute to neuronal pyroptosis–related changes.

**Nomenclature**
ASC: Apoptosis‐related spot–like protein containing caspase recruitment domain
BSA: Bovine serum albumin
Caspase‐1: Cysteinyl aspartate specific proteinase‐1
Cbl: Cobalamin
CCK‐8: Cell counting kit‐8
CNS: Central nervous system
DMEM: Dulbecco’s Modified Eagle Medium
ERK: Extracellular signal–regulated kinase
FBS: Fetal bovine serum
IL‐1β: Interleukin‐1 beta
KD: Knockdown
MAPKs: Mitogen‐activated protein kinases
MCM: Methylmalonyl‐CoA mutase
MMA: Methylmalonic acidemia
MMAcid: Methylmalonic acid
NF‐κB: Nuclear factor‐κB
NLRP3: NOD‐like receptor pyrin domain containing 3
OD: Optical density
OE: Overexpress
PCR: Polymerase chain reaction
SD: Standard deviation
siRNA: Small interfering RNA
TLR‐4: Toll‐like receptor 4
TNF‐α: Tumor necrosis factor‐α
VB: Viability.

## Author Contributions

Qiliang Li was responsible for the conception and design of the study. Shuqi Sun and Yumei Su performed the experiments and analyzed the data with the support of Lu Sun and Qiliang Li. Aihua Li, Lixin Hu, and Qiliang Li contributed reagents and materials. Shuqi Sun and Lu Sun wrote the paper.

## Funding

The work was supported by the National Natural Science Foundation of China (Grant 81802061), Science and Technology Innovation Development Project, Beijing Children’s Hospital, Capital Medical University, National Center for Children’s Health (Grant YN202401), and the Natural Science Foundation of Capital Medical University (Grant PYZ22147).

## Disclosure

All authors have reviewed and approved the final manuscript.

## Conflicts of Interest

The authors declare no conflicts of interest.

## Supporting Information

Additional supporting information can be found online in the Supporting Information section.

## Supporting information


**Supporting Information** Figure S1: mRNA expression of iNOS (M1 marker) and Arg1 (M2 marker) in BV2 microglia. C‐CTRL: untreated BV2 cells under normal culture conditions; C‐OE: BV2 cells overexpressing the ERK gene cultured under normal conditions; C‐KD: BV2 cells with ERK gene knockdown under normal culture conditions; M‐CTRL: BV2 cells treated with MMAcid for 3 days; and M‐OE: BV2 cells overexpressing the ERK gene treated with MMAcid for 3 days. M‐KD: BV2 cells with ERK gene knockdown treated with MMAcid for 3 days. Data are presented as mean ± SD (*n* = 3 biological replicates). ( ^∗^ *p* < 0.05 and  ^∗∗∗^ *p* < 0.001 compared to the control group). Figure S2: The expression levels of GSDMD‐N in HT22 neuronal cells with different treatments. C‐HT22: control group of HT22 cells cultured under normal conditions; M‐HT22: HT22 cells directly treated with methylmalonic acid; HT22/C‐BV2: HT22 cells cocultured with the supernatant of normal cultured BV2 cells; HT22/M3d‐BV2: HT22 cells cocultured with the supernatant of BV2 cells treated with methylmalonic acid for 3 days. Figure S3: Verification of ERK overexpression in BV2 microglial cells by lentiviral transduction. Representative bright‐field and fluorescence microscopy images of BV2 cells following lentiviral infection. Green fluorescence indicates expression of the reporter gene carried by the lentiviral vector, confirming successful transduction and supporting ERK overexpression in BV2 cells. Scale bar = 100 μm.

## Data Availability

Data will be made available upon request.
