# Supplementary material for: Mechanisms of Microglial NLRP3 Inflammasome Activation by Methylmalonic Acid in Methylmalonic Acidemia
Source: Mediators Inflamm. 2026 Jun 18;2026:1635641. doi: 10.1155/mi/1635641 (PMC13277775; doi:10.1155/mi/1635641)
Supplement: Supplementary file 1 — Supporting Information Figure S1: mRNA expression of iNOS (M1 marker) and Arg1 (M2 marker) in BV2 microglia. C‐CTRL: untreated BV2 cells under normal culture conditions; C‐OE: BV2 cells overexpressing the ERK gene cultured under normal conditions; C‐KD: BV2 cells with ERK gene knockdown under normal culture conditions; M‐CTRL: BV2 cells treated with MMAcid for 3 days; and M‐OE: BV2 cells overexpressing the ERK gene treated with MMAcid for 3 days. M‐KD: BV2 cells with ERK gene knockdown treated with MMAcid for 3 days. Data are presented as mean ± SD (n = 3 biological replicates). ( ∗ p < 0.05 and ∗∗∗ p < 0.001 compared to the control group). Figure S2: The expression levels of GSDMD‐N in HT22 neuronal cells with different treatments. C‐HT22: control group of HT22 cells cultured under normal conditions; M‐HT22: HT22 cells directly treated with methylmalonic acid; HT22/C‐BV2: HT22 cells cocultured with the supernatant of normal cultured BV2 cells; HT22/M3d‐BV2: HT22 cells cocultured with the supernatant of BV2 cells treated with methylmalonic acid for 3 days. Figure S3: Verification of ERK overexpression in BV2 microglial cells by lentiviral transduction. Representative bright‐field and fluorescence microscopy images of BV2 cells following lentiviral infection. Green fluorescence indicates expression of the reporter gene carried by the lentiviral vector, confirming successful transduction and supporting ERK overexpression in BV2 cells. Scale bar = 100 μm. [file MI-2026-1635641-s001.docx]

**Supplementary Materials**

Figure S1 mRNA expression of iNOS (M1 marker) and Arg1 (M2 marker) in BV2 microglia

C-CTRL: untreated BV2 cells under normal culture conditions; C-OE: BV2 cells overexpressing the ERK gene cultured under normal conditions; C-KD: BV2 cells with ERK gene knockdown under normal culture conditions; M-CTRL: BV2 cells treated with MMAcid for 3 days; M-OE: BV2 cells overexpressing the ERK gene treated with MMAcid for 3 days. M-KD: BV2 cells with ERK gene knockdown treated with MMAcid for 3 days. Data are presented as mean ± SD (n = 3 biological replicates). (**p* < 0.05, ****p* < 0.001 compared to the control group)


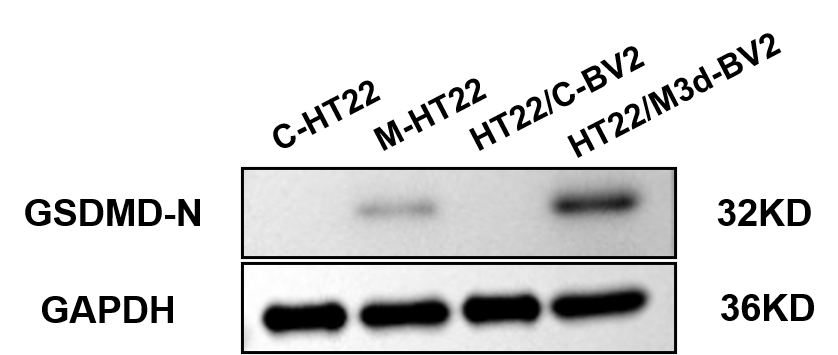


Figure S2 The expression levels of GSDMD-N in HT22 neuronal cells with different treatments.

C-HT22: Control group of HT22 cells cultured under normal conditions; M-HT22: HT22 cells directly treated with methylmalonic acid; HT22/C-BV2: HT22 cells co-cultured with the supernatant of normal cultured BV2 cells; HT22/M3d-BV2: HT22 cells co-cultured with the supernatant of BV2 cells treated with methylmalonic acid for 3 days.


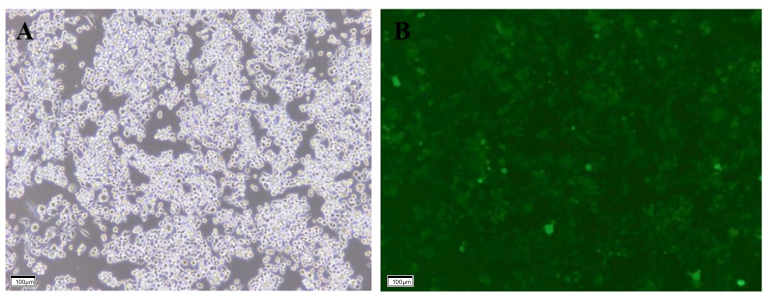


Figure S3 Verification of ERK overexpression in BV2 microglial cells by lentiviral transduction.
Representative bright-field and fluorescence microscopy images of BV2 cells following lentiviral infection. Green fluorescence indicates expression of the reporter gene carried by the lentiviral vector, confirming successful transduction and supporting ERK overexpression in BV2 cells. Scale bar = 100 μm.

.
